# School Quality and the Development of Cognitive Skills between Age Four and Six

**DOI:** 10.1371/journal.pone.0129700

**Published:** 2015-07-16

**Authors:** Lex Borghans, Bart H. H. Golsteyn, Ulf Zölitz

**Affiliations:** 1 Department of Economics and Research Centre for Education and the Labour Market (ROA), Maastricht University, Maastricht, the Netherlands; 2 Department of Economics, Maastricht University, Maastricht, the Netherlands; 3 IZA, Bonn, Germany and Department of Economics, Maastricht University, Maastricht, the Netherlands; University of Vienna, AUSTRIA

## Abstract

This paper studies the extent to which young children develop their cognitive ability in high and low quality schools. We use a representative panel data set containing cognitive test scores of 4-6 year olds in Dutch schools. School quality is measured by the school’s average achievement test score at age 12. Our results indicate that children in high-quality schools develop their skills substantially faster than those in low-quality schools. The results remain robust to the inclusion of initial ability, parental background, and neighborhood controls. Moreover, using proximity to higher-achieving schools as an instrument for school choice corroborates the results. The robustness of the results points toward a causal interpretation, although it is not possible to erase all doubt about unobserved confounding factors.

## Introduction

Abilities develop in a cumulative, dynamic process in which a child’s skills today determine tomorrow’s capabilities and potential to develop further cognitive skills. Cognition is formed relatively early in life and becomes less malleable as children age (see, e.g. [1]; Theoretical implications are discussed in [2]). Recent research has established that early differences in cognitive skills maintain or increase over time and thus are important predictors of later outcomes. [3] show that kindergarten test scores are highly predictive of later outcomes such as earnings, college attendance, and the quality of the attended college.

One essential question for public policy, therefore, is to what degree schools can contribute to the development of early cognitive skills. While there is evidence that preschool programs targeted at disadvantaged children (e.g., the Perry preschool program) have significant benefits, there is less literature on the role of school quality for children’s early cognitive development in a more general population of children.

This paper investigates the development of early cognitive skills in high and low quality schools using a new panel data set on a representative sample of school starters in Limburg, a southern province of the Netherlands. We relate the gains in cognitive tests scores measured over the course of two years to the quality of the school environment. We use the school’s average score on an achievement test, which children take at age 12 (grade 8), as an indicator for school quality.

Our results show that children in above-median quality schools develop their skills substantially faster than those in below-median quality schools. The difference in cognitive development is large and accumulates to 0.17 standard deviations by the end of grade two.

In our analyses, we control for many attributes which may drive the relationship between the quality of schools and cognitive development, and we use an instrumental variable approach to further investigate the extent to which the relationship is causal in nature. First and foremost, we control for initial cognitive ability in our regressions. This variable picks up selection into schools related to initial skills (cognitive ability) and should address concerns related to sorting. Our estimates show a low correlation between the quality of schools and this initial ability, indicating that selection of this kind does not seem to be a large problem in the Netherlands.

Secondly, since children’s cognitive development may also be affected by parental investments and socio-economic background, we control for the education levels of the mother and the father as well as household income. Parents with a higher socio-economic background may have stronger preferences for school quality and therefore be more likely to send their children to higher-achieving schools.

Third, since access to high quality schools may also be affected by neighborhood characteristics, we show that our results are robust to the inclusion of a set of neighborhood characteristics and neighborhood fixed effects. We include the percentage of households under the social minimum income, the percentage of households with low income (less than €25,100 per year), the percentage of households with high income (more than €46,500 per year) and the percentage of households with at least one child. This should pick up the heterogeneity of access at the postal code level and ensures that our results are not driven by a potentially non-random geographical distribution of school and student quality throughout different neighborhoods.

Although we control for a large set of important attributes, our results may still partly be driven by unobserved characteristics. In a robustness analysis, we estimate the relationships applying an instrumental variable approach. We use geographical proximity to a high-achieving school as an instrument for attending a school of higher quality. (This approach was originally suggested by [4] who applied the approach to estimate the returns to college education. Note that there is a difference between his and our approach, since college choices in the US might differ from primary education choice in the Netherlands for a number of reasons. The main identifying assumption in both cases is that distance to a good school in both setting is random. In this study we think this is less of a concern than in the US since there is limited segregation in the area of the Netherlands where we collected our data in comparison to the US context.) Proximity to schools plays a predominant role in the choice process of parents and is therefore highly related to the probability of choosing a school. Approximately 72 percent of the parents choose the primary school that is located closest to their residential location, and 93 percent of the parents choose one of the four schools closest to their residential location (a more elaborate analysis of the determinants of primary school choice is given in [5]). The results of this robustness analysis confirm our findings from the OLS estimation and provide point estimates similar to our baseline estimates. This indicates that our results appear to be causal in nature. However, the IV estimation hinges on the assumption that distance to a high quality school is unrelated to confounding factors. Sorting into neighborhoods is likely to be a minor concern for the area in the Netherlands where our data was collected since segregation is much less of a problem than in, for example, the US. Although in the Dutch context the assumption underlying the IV approach is therefore more likely to be valid than in the US, we remain cautious and abstain from drawing causal inferences throughout this paper.

Our analysis suggests that school quality appears to affect the development of cognitive skills of young children. This has important consequences since various studies have indicated that stimulating cognitive development at a young age can have benefits throughout life. For instance, analyses of the Perry preschool program and the STAR experiment reveal that interventions at the school level affect early child development and later outcomes. [6] and [3] use project STAR data to show that although the effect of the intervention on test scores and grades fades out after some time, the kindergarten test scores strongly correlate with wages at age 27. [3] find that a one percent increase in kindergarten test scores translates into a $94 increase in yearly earnings (after controlling for parental background). [7] study evidence from Germany and show that the development level of cognitive abilities at age two, four and eight are important predictors of later educational attainment and grades. [8] discusses how the quality of day care centers affects child outcomes. The authors conclude that high quality care has a positive and statistically significant association with child cognitive development. Our study is distinct from these studies in that we can relate the end of primary school aggregate test scores to the performance of children at the transition of preschool to primary school.

Our analysis also relates to the literature evaluating the effects of increased parental school choice possibilities on student achievement test scores. [9] investigate the effect of information provision on school choice and student achievement tests. The authors analyze the results of two experiments where the provision of information on school test scores leads to an increase in math and reading test scores in secondary education. In contrast to that, [10] do not find an effect of attending the “first choice” school on individual academic achievements when using the exogenous variation of high school admission lotteries in Chicago for their analysis.

The contribution of this paper is twofold. Firstly, whereas many studies have explored the effects of early intervention programs on outcomes, there is limited evidence on whether school quality affects the development process of children’s cognitive skills at the very beginning of primary school education. Some evidence related to our aim has been collected before, for instance by [11] who study the relationship between the quality of child care and child development through second grade. [12] who study role of pre-school education as a protective factor in the development of children who are at risk due to environmental and individual factors. On the other hand [13] argues that development of IQ and cognition is unrelated to systematic variation in quality of schools. Our results reveal that the effect of school quality appears to be already visible during the first year of preschool and continues into the second year. Secondly, our findings add to the literature on the formation of cognitive skills by showing that school quality appears to be an important environmental factor that is related to the development of cognitive skills at ages 4–6. Our results suggest that some parts of the later observed heterogeneity in student outcomes may already be established at an early stage of life due to differences in school quality. These findings are consistent with the results of [14] and [15], who underline the importance of early investments for the formation of skills.

The remainder of this paper is structured as follows. Section 2 provides background information on the Dutch education system. Section 3 discusses the data, and section 4 describes our identification strategy. Section 5 shows the results. Section 6 discusses robustness analyses, and section 7 concludes.

## The Dutch Primary Education System

Elementary education in the Netherlands consists of eight grades, and almost all children enter school at the age of four. Primary education is compulsory from the age of five, but all children can attend elementary schools from the age of four onwards. According to Statistics Netherlands, 98.5% of all children enroll in primary education at the age of four. Dutch parents enjoy the right to freely choose which elementary school their child will attend. Free school choice in the Netherlands was introduced more than 100 years ago. The results presented in this study can be interpreted as evidence from a school system that would be somewhat closer to a steady state of choice behavior than studies that investigate effects of recently introduced choice plans. If our results indicate that the school environment relates to the cognitive development at an early stage, it is unlikely that this relationship is driven by recent changes in educational policy. This complements the evidence from other regions that evaluates recently introduced policy changes in school choice. Dutch parents do not have to apply for schools nor face complex admission lotteries in the areas where our data was collected. There is also no default “home school” assignment or official choice constraints based on the residential location or school district. In addition, there are no school admission fees, and all public and private schools receive government funding if they fulfill certain requirements. Parents can freely choose a school that fits their educational or religious values. Parents can choose from public primary schools without a religious affiliation and Catholic, Protestant or Islamic elementary schools. Other schools like the Montessori, Jenaplan, or the union of Vrije scholen are dedicated to alternative educational concepts and offer different teaching methods. At the same time, the population density of the Netherlands is relatively high, and a number of elementary schools are usually available within walking or cycling distance. In the dataset that we use, parents have, on average, 6.2 primary schools to choose from within a radius of 2 kilometers. Capacity constraints do not play a major role for the choice process in the area where our data were collected. The most obvious costs that parents face when choosing a more distant school are the opportunity costs of time to travel to school and to classmates of their children.

The first two years of primary education (age 4–6) are comparable to kindergarten in the US. Children receive no formal schooling but engage in many preparatory activities. After completing the second preschool grade, the class composition and the schooling location usually remain the same. The fact that preschool and primary school in the Netherlands are de facto one institution allows us to directly link primary school quality indicators to preschool cognitive development. In grade eight, the last year of primary education (around age 12), children take a nationwide standardized achievement test, the CITO test. The result of the CITO test, together with the teacher’s recommendation determines which of three secondary school tracks a child will attend. Switching between the secondary school tracks becomes difficult as children age. Admission to scientific studies at university is only possible with a diploma from the highest secondary school track. This indicates that the CITO test is a high stakes achievement test. In the analysis, we use the school’s average CITO test score as an indicator for school quality.

## Methods and Data Description

The data used in this paper were collected in a cooperative project between elementary and secondary schools, school boards, municipalities and Maastricht University to analyze school performance in order to foster educational improvement. Student, parental, and school level data were collected in 2007 and 2008 in the southern part of the Dutch province Limburg. Out of the 216 primary schools in South Limburg, 210 took part in the study by providing their administrative data and distributing questionnaires to parents. An essential feature of this dataset is that test scores from school sources can be matched to detailed survey information from parents.

### 3.1 Ethics statement

Our study was approved by the Ethics Committee of the Faculty of Psychology and Neuroscience at Maastricht University (study protocol ECP-147ex/11-12-2014). The surveys we use were not specifically designed for the purpose of this study but are part of the earlier mentioned program which aims to monitor scholastic achievement of students. Prior to the survey conduction, the local school boards of Limburg approved the project and the questionnaire. The data were collected by the UM service unit MEMIC and were offered to us anonymized. The ethics committee approved the procedure for personal data protection.

The data used in this article contain sensitive information. The authors of this study are part of the economics of education group at Maastricht University which is the owner of the data. The schools participating in our study provided their administrative data and access to postal codes of homes and schools. The postal codes of parental housing locations are not freely available. These data are confidential in nature and cannot be made publicly available, since the 6 digit postal codes in the Netherlands can uniquely identify individual households. All interested researchers will be able to access the data for replication purposes upon request. (Applying to receive access to the confidential data can be done by contacting the corresponding author. Confidential parts of the data have to be analyzed in Maastricht. Alternatively, for replication purposes, researchers can submit Stata do-files which will be run for them by a member of the group. Codes needed for replication are available upon request. The restrictions related to data sharing and this procedure were approved by the Ethics Committee.

### 3.2 Data on preschool test scores

The central variable we use in our analysis to measure cognitive skills is the score on a test called “ordering test for kindergarten children.” (In Dutch: “ordenen toets voor kleuters”) In the first two years of education, four of these tests are conducted. The tests are designed to measure students’ progress during preschool and to identify possible problems in the cognitive development of children during the first two years of primary education. The test deals with matching and identification of shapes and objects as well as concepts like size, amount or length. Every test contains 42 questions. Examples of the test questions can be found in (S1 Text). Relative to standard adult IQ tests, this test is likely to be less precise. Because the tests are the dependent variable in our estimations, if the test is measuring skills imprecisely, this will decrease the efficiency but not the consistency of our point estimates. Note that, measurement error would only attenuate our estimates when tests are used as independent variables. This is the case when we control for test 1. Measurement error in test 1 will attenuate its coefficient meaning that we are underestimating the impact of test 1. This could to some degree explain the finding that initial cognitive ability does not significantly relate to school choice.

Tests one and two contain the same questions, while the items in tests three and four are also the same but different from the previous two tests. Children had, on average, six months of schooling when taking the first test. The average time that elapsed between the first two tests in grade one of preschool is 4.3 months, and there are 3.9 months between tests three and four in grade two of preschool. The average time that passed between test one and four is 16.3 months.

**Fig 1:**
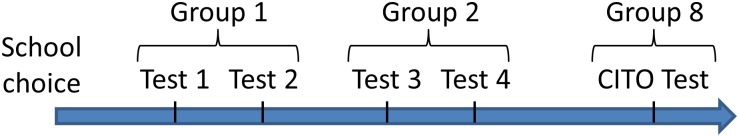
Timeline of the conducted tests in preschool. **Note:** Tests 1 was taken in January 2007. Test 2 was taken in May or June 2007. Test 3 was taken in January 2008, and Test 4 was taken in May or June 2008. Tests one and two contain the same questions. Tests three and four also contain the same items. Children had, on average, six months of schooling when taking the first test. The average time that elapsed between the first two tests in grade one of preschool is 4.3 months, and 3.9 months passed between tests three and four in grade two of preschool. The average time that passed between tests one and four is 16.3 months.

All four tests are conducted by elementary school teachers who have received detailed instructions. The teachers read the questions aloud, and children mark the correct answer in their test booklet. Within a given school, most children take each test on one specific day. Fig 1 shows a timeline of the conducted tests during preschool. The first test in grade one of preschool usually takes place in January and is repeated with the exact same questions in May or June (second test). The third test is usually conducted in January of the second preschool grade and is then repeated with the same content in May or June (fourth test). The exact date of testing serves as an additional control in the later analysis where we account for early and postponed testing dates and the time that elapsed between two tests. We control for the time that passed between the respective tests to take into account that some children may have had more time to acquire cognitive skills.

The participation was voluntary, and there were no financial or other incentives related to the test performance of the children present. In total, 162 schools out of the 210 participating schools decided to conduct the proposed test for cognitive development and submitted student data in 2007 and/or in 2008. Within the participating schools, 85% of the children in grade one and two took part in at least one of the tests. At the school level, higher-performing schools had about 2 percentage points higher participation rates.

During 2007 and 2008, a total of 3,225 first graders participated in test one and test two. Children had, on average, 6.0 months of school experience when they were tested first. The average age on the day of the first testing was 4.5 years. As for second graders, 2,665 participated in tests 3 and 4. In the 2007 wave, we observe the test scores of 1,463 first graders and 134 second graders. In the 2008 wave, we observe the test scores of 1,762 first graders and 2,531 second graders. Out of the 1,463 first graders who took the tests in the 2007 wave, we can follow 1,112 students throughout all four consecutive tests. We do not find evidence for selective attrition on observables over time.

**Fig 2:**
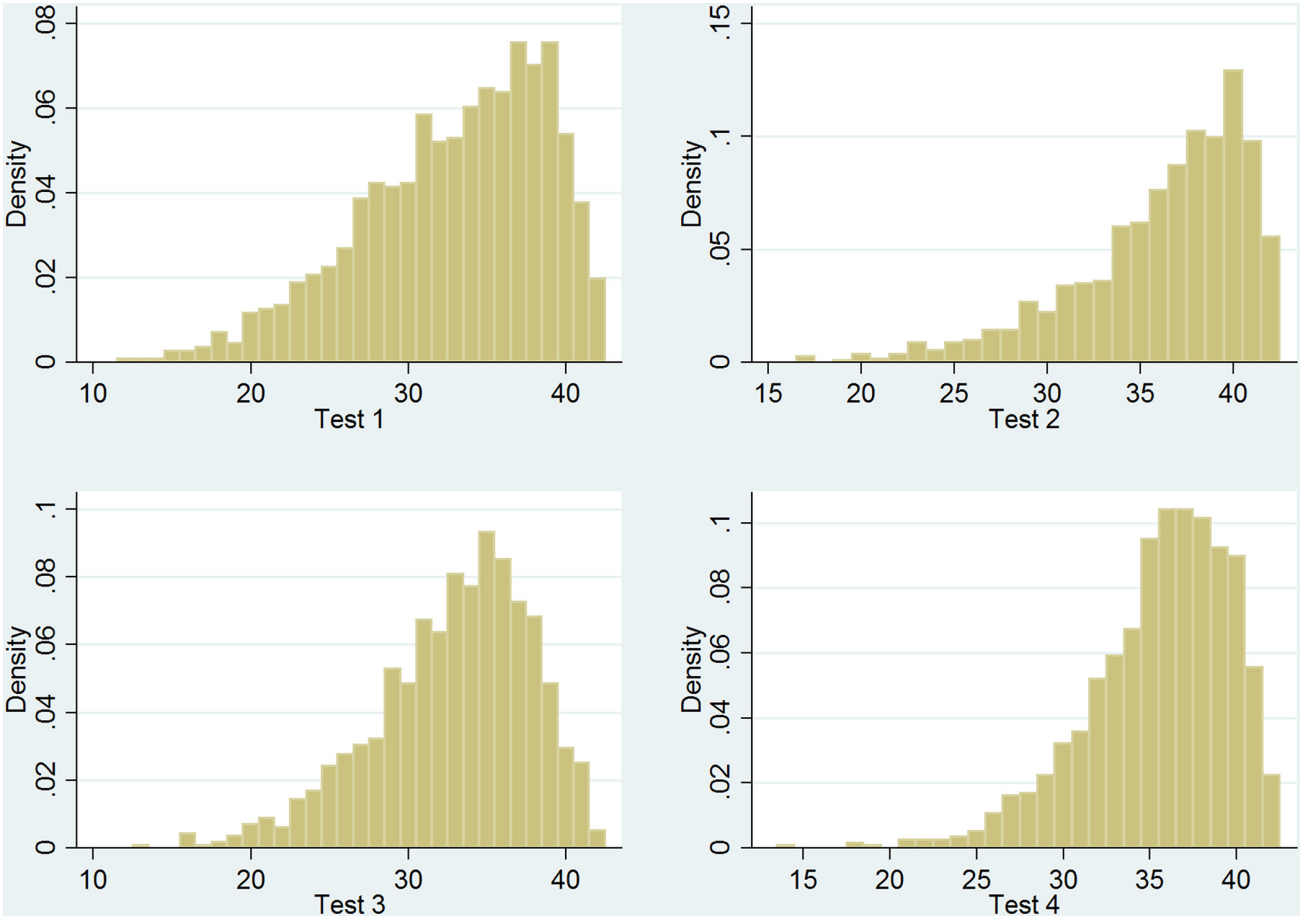
Cognitive development during preschool: Distribution of test scores. **Notes:** Test two is a repetition of the first test containing the same questions. Likewise, test four is the same as test three. Children had, on average, six months of schooling when taking the first test. The average time that elapsed between the first two tests in grade one of preschool is 4.3 months, and 3.9 months passed between tests three and four in grade two of preschool. The time that passed between test one and four is 16.3 months. n = 1299.

Fig 2 shows the distribution of outcomes for the four different tests. The individual scores range between 0 and 42. For the repeated tests (tests two and four) the distributions are shifted to the right. The distribution of test 2, which was taken at the end of grade one of preschool, shows that the data is censored to some extent. Five percent of all students reached the maximum number of 42 points. Children at better schools have a higher measured initial ability, and therefore the potential gains in the measured cognitive development are somewhat smaller. Due to this ceiling effect, we may underestimate the relationship between school quality on cognitive development to some degree. For all regressions we report in this paper, we have standardized tests 1 to 4 over the estimation sample to mean zero and unit standard deviation.

Observing the cognitive achievements of a child repeatedly over a time span of two years makes our data unique and allows us to relate the rate of cognitive development to the characteristics of the school that the child attends. The repeated measures also enable us to control for the previous state of development and to obtain better estimates of the added value of school quality.

### 3.3 School performance measure

**Fig 3:**
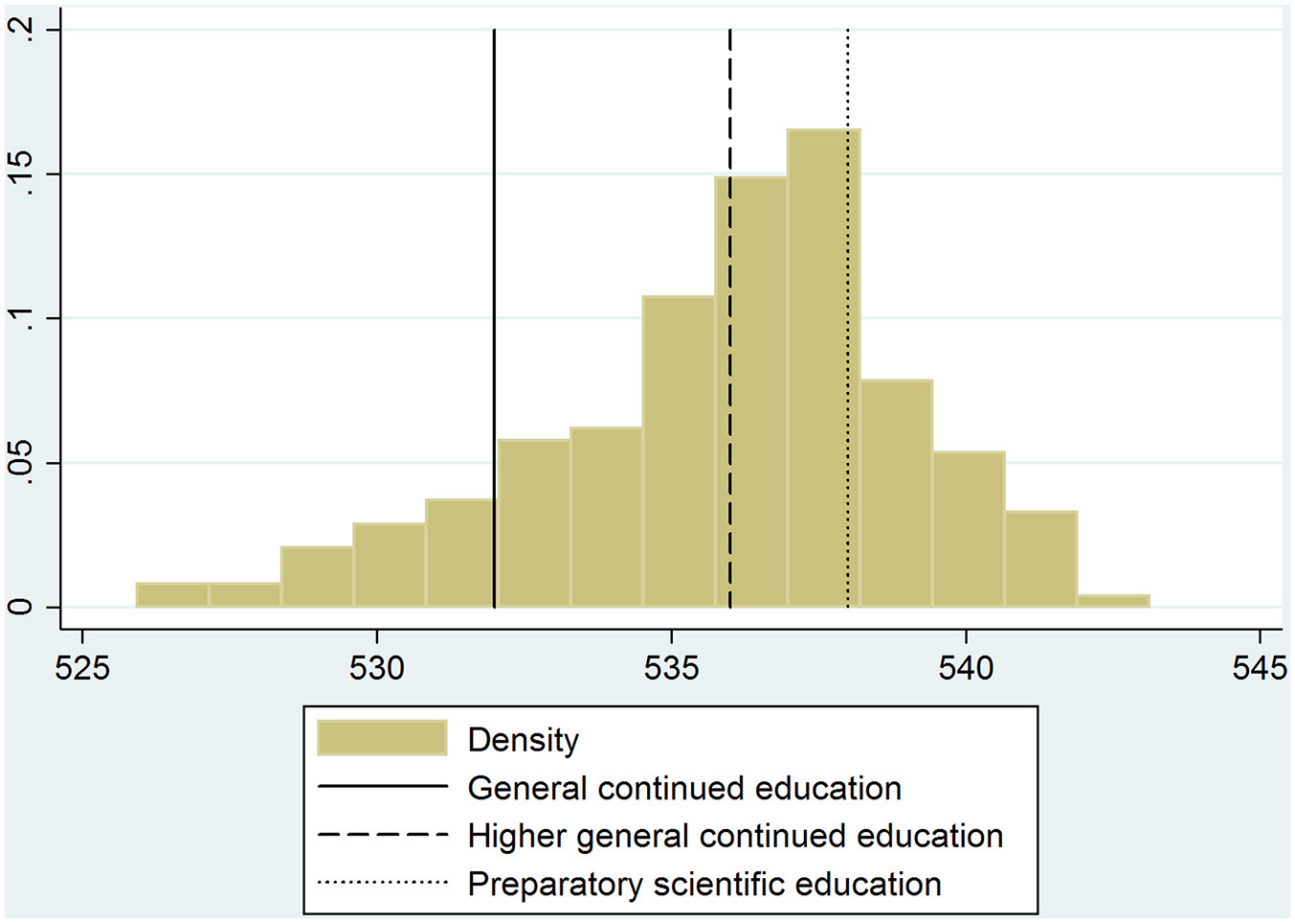
Distribution of the school quality measure. **Notes:** These are school level averages of nation-wide end of primary education achievement tests (CITO scores). The CITO test is designed to have a score ranging from 500 to 550 for each individual student. The vertical lines mark the recommended thresholds for secondary school admission. The figure shows the distribution of the school level three year average of the CITO scores from 2008, 2009 and 2010.

We use the school’s average CITO score as a measure for the quality of the school. This is a standardized test, which is conducted nationwide at specific days at the end of primary education. It is used for tracking students moving into secondary education. The score ranges between 500 and 550 at the individual level. Because school quality may fluctuate from year to year to some extent, we take a three year average CITO test score as our measure. Fig 3 shows the distribution of the three year school average CITO scores of the schools that took part in the program, as well as the recommended secondary school tracking thresholds.

The school’s average CITO score is potentially influenced by many different school attributes—the quality of teachers, the educational concept or pedagogy of the school and the technical equipment—but also the composition, ability, motivation and parental background of the children that graduate from this school are potential components of this score. In this paper, we are not primarily interested in what is causing a high or low CITO score. We aim to answer whether it is beneficial for the early cognitive development of a child to choose and attend a school that performs well on this indicator of the quality of school environment.

Throughout the analysis, we will use a binary indicator for the quality of the school environment. Based on the three year overall school’s average CITO score, we label all schools that have an above median score as “higher-achieving” and schools below median as “lower-achieving.” This arbitrarily defined threshold simplifies the analysis and interpretation of the coefficients. The obtained results can be interpreted as the effect of attending an above-median (higher-achieving) school versus attending a below-median school on the cognitive development of a child. All the main results of this paper remain qualitatively similar when using a continuous version of the CITO score in the estimations (see S7 and S8 Tables).

The summary statistics in Table 1 show how the schools in our dataset differ in a number of observable characteristics when split at the median into higher- and lower-achieving schools. Children at higher-achieving schools have, on average, higher educated parents, come from a household with more income, and perform better on all four cognitive ability tests during preschool. This table can be interpreted as evidence for selection into schools on observable characteristics and raises valid concerns about potential differences in unobservable dimensions.

**Table 1:** Summary statistics.

	(1)		(2)		(3)		(4)	
	All students		All fours tests sample		Higher-achieving schools		Lower-achieving schools	
Preschool test-scores for cog. development								
Test score 1 (grade 1)	32.38	(6.23)	32.40	(6.23)	33.25	(5.88)	31.45	(6.47)
Test score 2 (grade 1)	35.30	(4.83)	35.86	(4.83)	36.50	(4.36)	35.16	(5.22)
Test score 3 (grade 2)	32.62	(5.02)	32.84	(5.02)	33.70	(4.64)	31.89	(5.26)
Test score 4 (grade 2)	35.00	(4.16)	35.56	(4.16)	36.25	(3.82)	34.80	(4.4)
Age at the time of school start	3.96	(0.31)	3.97	(0.31)	3.96	(0.31)	3.99	(0.32)
Months between test 1 and 2	4.34	(1.07)	4.22	(1.07)	4.27	(0.82)	4.16	(1.29)
Months between test 2 and 3	8.24	(2.60)	8.26	(2.6)	8.03	(2.19)	8.52	(2.97)
Months between test 3 and 4	3.87	(1.77)	4.04	(1.77)	4.12	(1.65)	3.94	(1.89)
One if pupil female	0.48	(0.50)	0.48	(0.50)	0.50	(0.50)	0.45	(0.50)
Distribution of household income in Euro								
below 800	2.70%		2.10%		0.73%		3.93%	
800–1250	9.90%		10.50%		7.58%		14.43%	
1250–1750	8.60%		10.50%		8.31%		13.44%	
1750–2250	12.70%		13.45%		11.25%		16.39%	
2250–2750	18.20%		15.69%		16.38%		14.75%	
2750–3250	17.50%		17.65%		19.56%		15.08%	
3250–3750	11.60%		9.94%		11.25%		8.20%	
3750–4250	6.10%		6.30%		8.07%		3.93%	
4250–4750	3.60%		3.92%		5.38%		1.97%	
4750–5250	2.70%		4.06%		4.89%		2.95%	
above 5250	6.40%		5.88%		6.60%		4.92%	
Education level mother (highest obtained degree):								
No degree	5.10%		5.83%		2.89%		9.68%	
Lower vocational education	7.90%		7.70%		5.77%		10.22%	
General continued education	11.40%		11.32%		9.90%		13.17%	
Preparatory scientific education	43.40%		47.84%		47.84%		47.85%	
Higher professional education	23.90%		20.19%		25.57%		13.17%	
University degree	8.40%		7.12%		8.04%		5.91%	
Education level father (highest obtained degree):								
No degree	5.40%		6.49%		4.40%		9.30%	
Lower vocational education	9.60%		10.82%		9.22%		12.96%	
General continued education	10.00%		8.77%		6.71%		11.55%	
Preparatory scientific education	35.80%		36.78%		36.90%		36.62%	
Higher professional education	26.80%		24.88%		28.30%		20.28%	
University degree	12.40%		12.26%		14.47%		9.30%	
3 year school average CITO score	536.10		536.38		538.43		533.92	
	(3.07)		(2.92)		(1.54)		(2.23)	
Number of schools	156		87		48		39	
Number of students	7681		1299		685		614	
Average no. of students per school	49.24		14.93		14.27		15.74	

**Notes:** Since we do not know the exact date of the first day at school from administrative sources, we use the questionnaire answer to the question, “*In which month did your child go to school for the first time*?” to calculate the variable “Months at school at time of test 1”. Standard deviations are in parentheses.

Table 2 shows the correlations between the four different tests. As mentioned earlier, test 1 and 2 are the same, and test 3 and 4 are also the same. Scores on test 1 and 2 are highly correlated (0.71), and scores on test 3 and 4 are also highly correlated (0.69), as expected, but a substantial amount of variation remains. The correlations are lower between the two sets of the tests (around 0.55). These correlations in part reflect noise in the answers but may also give an indication that children differ in their developmental trajectories.

**Table 2:** Pairwise correlations between the four test scores.

	Test 1	Test 2	Test 3	Test 4
Test 1	1			
Test 2	0.7119***	1		
Test 3	0.5456***	0.5653***	1	
Test 4	0.5446***	0.5602***	0.6944***	1

*** p<0.01

### 3.4 Data from the parental questionnaire

All parents with children in grades one, two or three in the participating schools received a questionnaire via the schools. Children took the questionnaires home and brought them back to school in a sealed envelope after their parents had filled them out.

The questionnaire contained 51 questions with more than 150 items. Important for the purpose of this paper are the education level of the parents, household income and the geographical location of their home, which allows us to determine which postal code attributes apply and to calculate the distance to schools. The questionnaire further assessed motives for school choice, school satisfaction and the parents’ expectations about future child performance and outcomes.

## Empirical Strategy

This paper investigates how school quality, measured as the average CITO test score at the end of primary school, relates to the rate of cognitive development at the beginning of primary education. In our main specification, we run OLS regressions in which we control for important potential confounders which may drive the relationship between school quality and cognitive development.

Firstly, in all specifications, we control for scores on test 1 to correct for differences in the initial level of cognitive ability. This will pick up potential selection of high ability children into high quality schools. Regressing this baseline measure of cognitive ability on the quality of the chosen school reveals limited evidence of such selection. In order to investigate whether high ability students select into high-achieving schools, we perform an OLS regression where we regress baseline cognitive ability on the quality of the chosen school (see S1 Table. The dependent variable represents the standardized values of test 1, which was taken after, on average, 6 months of schooling. Column (1) shows that children at higher-achieving schools perform, on average, 0.173 standard deviations higher than children at below-median schools. When including the education level of both parents and the aggregate household income as controls in column (2), the coefficient decreases to 0.112 standard deviations but remains significant at the 5 percent level. In column (3), when taking into account differences in neighborhood characteristics, the coefficient drops further and becomes insignificant. This indicates that after controlling for these variables, we no longer find evidence for selection into schools based on initial ability.

We also control for the time that passed between the respective tests to take into account that some children may have had more time to acquire cognitive skills. Note that, instead of controlling for test 1, we could also take the difference between the test scores as a dependent variable. Applying this approach provides identical results.

Secondly, parental preferences for schools may be heterogeneous across different socio-economic subgroups. For instance, parents with stronger preferences for school quality may have children with higher initial cognitive abilities and a steeper development trajectory. In order to take this into account, we control for the education level of the mother and the father as well as household income.

Thirdly, children from higher socioeconomic backgrounds may have better access to high-quality schools due to their residential location, i.e., the clustering of higher-achieving schools in advantaged neighborhoods. We address the issue of geographical clustering by including a number of neighborhood characteristics at the postal code level. These neighborhood controls are the percentage of households under the social minimum income, the percentage of households with low income (less than €25,100 per year), the percentage of households with high income (more than €46,500 per year), and the percentage of households with at least one child.

In a robustness analysis, we use distance to a high-achieving school as an instrument. This instrument has a substantial influence on the decision of choosing a higher- or lower-achieving school and is plausibly unrelated to confounding factors that drive cognitive development.

## Results

Table 3 shows the basic estimation results using an OLS regression. We regress scores on tests 2, 3, and 4 on a dummy variable which indicates whether the child attends a high-quality school. Panel A shows the results without additional family or neighborhood controls. In Panel B, we show how the inclusion of parental background controls and neighborhood controls alters our point estimates. Columns 1, 2 and 3 show the effect without any additional controls. In columns 4, 5 and 6, we control for the initial level of cognitive skills and the time between the two tests. The estimates point to a positive relationship between attending a high-quality school and cognitive development. Comparing columns 4, 5 and 6 in Panel A reveals that the size of the coefficient “Higher-achieving school” increases for every consecutive test taken. While the effect of school quality on test 2 is ambiguous, there is a clear increase for test 3 and test 4. The effect increases over time both in size and significance.

**Table 3:** Attending a higher-achieving school and cognitive development—OLS estimation.

**Panel A**	(1)	(2)	(3)	(4)	(5)	(6)
	Test 2	Test 3	Test 4	Test 2	Test 3	Test 4
Higher achieving school	0.185***	0.330***	0.313***	0.066*	0.212***	0.213***
	(0.049)	(0.055)	(0.048)	(0.035)	(0.045)	(0.039)
Test 1				0.686***	0.618***	0.539***
				(0.020)	(0.026)	(0.023)
Time between test 1 & 2 (in months)				0.001		
				(0.024)		
Time between test 1 & 3 (in months)					0.060**	
					(0.026)	
Time between test 1 & 4 (in months)						0.039**
						(0.017)
Constant	0.062*	-0.127***	-0.058*	0.074	-0.826***	-0.661**
	(0.036)	(0.040)	(0.035)	(0.097)	(0.313)	(0.270)
Observations	1,112	1,112	1,112	1,112	1,112	1,112
Adj. R-squared	0.0116	0.0302	0.0361	0.522	0.359	0.365
Parental background controls	No	No	No	No	No	No
Neighborhood controls	No	No	No	No	No	No
**Panel B**	(7)	(8)	(9)	(10)	(11)	(12)
	Test 2	Test 3	Test 4	Test 2	Test 3	Test 4
Higher achieving school	0.040	0.163***	0.177***	0.056	0.153***	0.167***
	(0.036)	(0.046)	(0.040)	(0.037)	(0.048)	(0.041)
Test 1	0.671***	0.599***	0.521***	0.673***	0.598***	0.519***
	(0.020)	(0.027)	(0.023)	(0.020)	(0.027)	(0.023)
Time between test 1 & 2 (in months)	0.001			-0.004		
	(0.024)			(0.024)		
Time between test 1 & 3 (in months)		0.051*			0.050*	
		(0.027)			(0.027)	
Time between test 1 & 4 (in months)			0.035**			0.038**
			(0.017)			(0.017)
Constant	0.078	-0.756**	-0.645**	0.236	-0.406	-0.086
	(0.100)	(0.315)	(0.270)	(0.331)	(0.481)	(0.437)
Observations	1,112	1,112	1,112	1,112	1,112	1,112
Adj. R-squared	0.528	0.366	0.379	0.528	0.368	0.381
Parental background controls	Yes	Yes	Yes	Yes	Yes	Yes
Neighborhood controls	No	No	No	Yes	Yes	Yes

**Notes:** All test scores are standardized to mean zero and a standard deviation of one. A higher-achieving school is defined as having an above median three year school average CITO score. Parental background controls are the household income and the education level of the father and mother. Neighborhood controls include a set of variables measured at the four digit postal code area. The neighborhood controls are the percentage of households under the social minimum income, the percentage of households with low income (less than €25,100 per year), the percentage of households with high income (more than €46,500 per year) and the percentage of households with at least one child. The data on neighborhood characteristics was collected by CBS Statistics Netherlands. S2 Table shows the full model and reports the coefficients and standard errors for all included controls. Standard errors are in parentheses;

*** p<0.01,

** p<0.05,

* p<0.1.

In columns 10–12 of Panel B Table 3, we add a set of neighborhood controls to the model. These include the percentage of households under the social minimum income, the percentage of households with low income (less than €25,100 per year), the percentage of households with high income (more than €46,500 per year) and the percentage of households with at least one child at the postal code level of the school. Children at higher-achieving schools develop cognitive abilities faster during school. The effect remains similar when including parental background characteristics and the above mentioned neighborhood controls in Panel B. In the most conservative model in column 12, children at above median schools perform 0.17 standard deviations higher on test 4 compared to children at lower-achieving schools. Holding everything else constant, this means that children attending above median schools answer, on average, 1.1 more questions correctly (out of the 42 question in test 4). Test 4 was conducted, on average, after 22.1 months of schooling and 16.1 months after taking test 1. Given this time span, the quality of the schools seems to be strongly related to the cognitive skill development of the four to six year olds. We have also tested whether these effects are different for boys and girls. S4 Table shows that we find no statistically significant gender difference. Reasons for the included neighborhood controls not to add much to the explanatory power of the model might be the relatively low degree of segregation in the Dutch province where we collected our data, or the fact that parental background controls already capture most variation in test scores unrelated to school quality. A different explanation may be that the postal code area is a too large unit to capture every-day life neighborhood characteristics. If this is the case, our results may to some extent be subject to endogeneity due to unobserved neighborhood characteristics.

**Fig 4:**
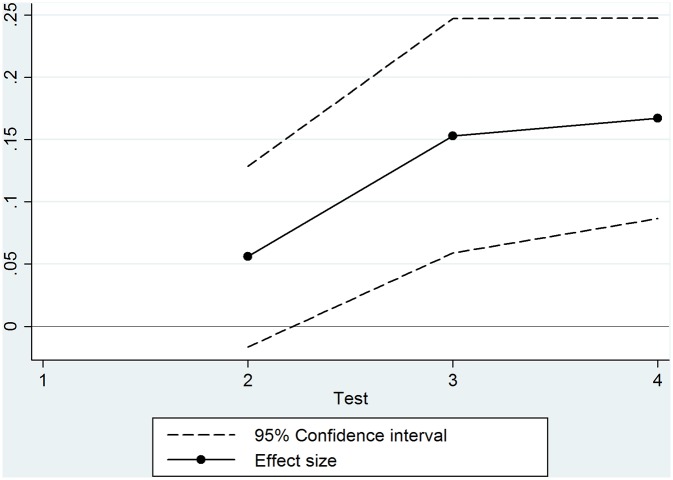
The relationship between attending a higher-achieving school and cognitive test scores. **Notes:** The figure shows the size of the relationship obtained from the regressions in Table 3 (columns 4, 5, 6).

Fig 4 illustrates how the size of the relationship grows over time. On the horizontal axis, we plot the average number of months that children have been at school when they were tested. The vertical axis shows the size of the relationship between “attending a higher-achieving school” and cognitive development. The increase in the size of the relationship suggests that the cognitive skills of children at lower- and higher-performing schools follow different growth patterns. Whether these differences at age six further increase, stabilize or decrease after the first two years is not clear from the time horizon of our data.

In Table 4, we investigate whether the relationship between attending a higher-achieving school and cognitive development is heterogeneous with respect to the baseline ability by including an interaction term between the first test and the variable “Higher-achieving school”. We find no evidence for heterogeneous effects in this estimation. Children with lower initial levels of cognitive ability do not seem to benefit more from being in the environment of a higher-achieving school. S5 Table provides some evidence that non-response to the survey does not bias the results. The table shows our main regression without controls for both the survey and the full sample. Point estimates reported here are not significantly different from each other.

**Table 4:** Heterogeneous relationships—OLS estimation.

	(1)	(2)	(3)	(4)	(5)	(6)
	Test 2	Test 3	Test 4	Test 2	Test 3	Test 4
Higher achieving school	0.044	0.165***	0.183***	0.060	0.155***	0.171***
	(0.036)	(0.047)	(0.040)	(0.037)	(0.048)	(0.041)
Higher achieving school * Test 1	-0.057	-0.027	-0.074	-0.055	-0.030	-0.071
	(0.040)	(0.052)	(0.045)	(0.040)	(0.053)	(0.045)
Test 1	0.699***	0.613***	0.559***	0.701***	0.613***	0.555***
	(0.029)	(0.037)	(0.032)	(0.029)	(0.037)	(0.032)
Time between test 1 & 2 (in months)	-0.002			-0.007		
	(0.024)			(0.024)		
Time between test 1 & 3 (in months)		0.050*			0.049*	
		(0.027)			(0.027)	
Time between test 1 & 4 (in months)			0.034**			0.038**
			(0.017)			(0.017)
Constant	0.089	-0.748**	-0.634**	0.223	-0.410	-0.106
	(0.100)	(0.315)	(0.270)	(0.331)	(0.482)	(0.437)
Observations	1,112	1,112	1,112	1,112	1,112	1,112
Adj. R-squared	0.528	0.366	0.380	0.529	0.368	0.382
Parental background controls	Yes	Yes	Yes	Yes	Yes	Yes
Neighborhood controls	No	No	No	Yes	Yes	Yes

**Notes:** All test scores are standardized to mean zero and a standard deviation of one. A higher-achieving school is defined as having an above median three year school average CITO score. Parental background controls are the household income and the education level of the father and mother. Neighborhood controls include a set of variables measured at the four digit postal code area. The neighborhood controls are the percentage of households under the social minimum income, the percentage of households with low income (less than €25,100 per year), the percentage of households with high income (more than €46,500 per year) and the percentage of households with at least one child. The data on neighborhood characteristics was collected by CBS Statistics Netherlands. S3 Table shows the full model and reports the coefficients and standard errors for all included controls. Standard errors are in parentheses;

*** p<0.01,

** p<0.05,

* p<0.1.

## Robustness Analyses

Although we control for a large set of important attributes in our regressions, our results may still partly be driven by unobserved characteristics. Our proposed solution is to apply an instrumental variable approach using proximity to high-quality schools as an instrument. This approach was originally introduced by Card (1995) [4] who applied the approach to estimate the returns to college education. This instrument should have a substantial influence on the decision of choosing a higher- or lower-achieving school but should be unrelated to confounding factors that drive cognitive development. S9 Table shows how the instrument correlates with student observables.

The first stage regressions in Table 5 show how the choice of a high-quality school relates to various measures of distance from a school to the residential location of the student. The dependent variable “Attending higher-achieving school” is an indicator variable that equals one if the child attends an above median school. Column 1 shows that parents are less likely to choose a high-quality school if it is located further away. People live at various distances to schools; therefore, instead of using the distance in kilometers to a high-quality school, it is more precise to ask whether the closest school is a high-performing school. We use as an instrument in our IV estimation the proximity dummy included in column 2, which equals one if the closest school, given the residential location of the student, belongs to the category “higher-achieving”. The estimates show that this is a strong instrument for attending a higher-achieving school. Our results also hold when we use distance in kilometers as an instrument. We use the proximity to a high-achieving school as an instrument because, as the F-statistics in the table indicate, it is a much stronger instrument than distance in kilometers. Column 3 shows that including family characteristics, such as the education level of the mother and father as well as household income, does not affect the coefficient of the instrument. These covariates are largely unrelated to the probability of attending a higher-achieving school. Column 4, the full model, which is the first stage in our later IV estimations, includes the initial ability test—our baseline measure for cognitive ability—and shows that children with a higher performance on test 1 are somewhat more likely to attend an above median school. The inclusion of the scores on test 1 does not add much to the explanatory power of the model. As expected, the proximity coefficient remains virtually unchanged.

**Table 5:** First stage regressions—Determinants of attending higher-achieving schools.

	(1) Attending higher- achieving school	(2) Attending higher- achieving school	(3) Attending higher- achieving school	(4) Attending higher- achieving school
Distance to the next higher-achieving school (km)	-0.098***			
	(0.021)			
One if closest school higher-achieving		0.741***	0.731***	0.729***
		(0.020)	(0.021)	(0.021)
Education level mother middle			0.065	0.061
			(0.041)	(0.041)
Education level mother high			0.066	0.057
			(0.048)	(0.048)
Mother education level missing			0.159**	0.160**
			(0.074)	(0.073)
Education level father middle			0.034	0.033
			(0.039)	(0.039)
Education level father high			0.025	0.024
			(0.043)	(0.043)
Father education level missing			-0.070	-0.072
			(0.068)	(0.068)
Income 1501–2500 €			-0.004	-0.008
			(0.050)	(0.050)
Income 2501–3500 €			-0.008	-0.012
			(0.049)	(0.049)
Income 3501–4500 €			0.025	0.019
			(0.057)	(0.057)
Income above 4501			0.023	0.020
			(0.059)	(0.059)
Income information missing			-0.037	-0.043
			(0.049)	(0.049)
Test 1				0.020*
				(0.012)
Constant	0.459***	0.119***	0.057	0.065
	(0.020)	(0.015)	(0.048)	(0.048)
Observations	1,112	1,112	1,112	1,112
F statistic excluded instrument(s)	21.16	1334.82	1213.09	1208.52
Adj. R-squared	0.0178	0.546	0.546	0.547

**Notes:** The dependent variable “Attending higher-achieving school” is defined as having an above median three year school average CITO score. The omitted education categories are “Education level mother low” and “Education level father low”. The omitted monthly household income category is “Income below €1,500”. Standard errors are in parentheses;

*** p<0.01,

** p<0.05,

* p<0.1.

The IV approach relies on the assumption that proximity to higher-achieving schools is a strong predictor of choice and that it is unrelated to the error term in the equation explaining the cognitive ability test score, conditional on including parental background and neighborhood characteristics. Concerning the first condition, Table 5 shows that the F-statistic of the instrument is very high, which suggests that the instrument is strong.

Proximity to an above median performing school must also be exogenous. Estimates are potentially biased if school quality is not randomly distributed in a geographical sense. This may be the case if people choose their housing location based on the similarity of neighborhood characteristics or if families with a higher socioeconomic background simply prefer living closer to higher-achieving schools. Reversing the causality, “good” schools may develop in neighborhoods with a higher socio-economic status. “Lower-achieving” schools might have a lower status because they serve the population of a less advantaged neighborhood. In addition to that, children with higher cognitive ability might be born closer to better schools.

Our strategy relies on the assumption that proximity to an above median school is random, i.e., that it is uncorrelated to unobserved determinants of children outcomes. However, this is not obvious: there is at least anecdotal evidence that parents take school quality into account when choosing a place to live. Consistent with this evidence, the literature has found an impact of school quality on housing prices [16] [17]. This might especially be true for primary schools, where parents may not want to commute and children are too young to commute by themselves.

We cannot directly test for the instrument’s exogeneity, but we address this issue by (1) controlling for initial ability and (2) controlling for a set of neighborhood characteristics at the postal code level of the school, which may pick up the effects of non-random variation in the proximity to higher-quality schools. Sorting into neighborhoods is likely to be a minor concern for the area in the Netherlands where our data was collected since segregation is much less of a problem than in, for example, the US. This claim is supported by the fact that the inclusion of neighborhood controls in our estimations adds little to the explanatory power of our models.

Table 6 shows the estimation results we obtain from IV regressions in which the dependent variables are the outcomes of three cognitive ability tests taken during the first two years of school. In the full model in column 6, the estimated coefficient is 0.22, which shows that, if anything, the results from our OLS estimations are somewhat underestimated. (S8 Table shows that these results hold when using a continuous school quality indicator.) The fact that the coefficient remains positive and significant (even increases) when we control for many potentially important observables and use an IV approach strengthens the case for a causal interpretation of our estimates. It is implausible that unobserved factors could drive down the results in any substantial way. However, we remain cautious to draw strong causal inferences.

**Table 6:** Attending a higher-achieving school and cognitive development—IV estimation.

	(1)	(2)	(3)	(4)	(5)	(6)
	Test 2	Test 3	Test 4	Test 2	Test 3	Test 4
Higher-achieving school	0.063	0.225***	0.231***	0.093*	0.212***	0.218***
	(0.049)	(0.064)	(0.054)	(0.052)	(0.066)	(0.057)
Test 1	0.670***	0.597***	0.519***	0.672***	0.597***	0.518***
	(0.020)	(0.026)	(0.023)	(0.020)	(0.026)	(0.023)
Time between test 1 & 2 (in months)	-0.002			-0.009		
	(0.024)			(0.024)		
Time between test 1 & 3 (in months)		0.047*			0.048*	
		(0.026)			(0.027)	
Time between test 1 & 4 (in months)			0.034**			0.037**
			(0.017)			(0.017)
Constant	0.080	-0.745**	-0.645**	0.246	-0.396	-0.074
	(0.098)	(0.311)	(0.267)	(0.326)	(0.475)	(0.431)
Observations	1,112	1,112	1,112	1,112	1,112	1,112
Parental background controls	Yes	Yes	Yes	Yes	Yes	Yes
Neighborhood controls	No	No	No	Yes	Yes	Yes

Notes: The instrumented variable is “Higher-achieving school”. The sample is restricted to the children who took all four consecutive tests and started school in 2007. All test scores are standardized to mean zero and a standard deviation of one. A higher-achieving school is defined as having an above median three year school average CITO score. Parental background controls are the household income and the education level of the father and mother. Neighborhood controls include a set of variables measured at the four digit postal code area. The neighborhood controls are the percentage of households under the social minimum income, the percentage of households with low income (less than €25,100 per year), the percentage of households with high income (more than €46,500 per year) and the percentage of households with at least one child. The data on neighborhood characteristics was collected by CBS Statistics Netherlands. Standard errors are in parentheses; S6 Table shows the full model and reports the coefficients and standard errors for all included controls. S7 Table shows the same model using a continuous quality indicator.

*** p<0.01,

** p<0.05,

* p<0.1.

## Conclusion

In this paper, we show that children who attend schools with higher average scores (on achievement tests in grade six) develop their cognitive skills faster than those in lower achieving schools during the first two years of school. This observed difference in developed skills is substantial even when controlling for a baseline measure of cognitive skills.

Our results confirm previous evidence that underlines the importance of school choice and school quality for cognitive development and later outcomes [9] and; [18]. We show that the school-related development differences are substantial and are already visible as early as ten months after the start of preschool. School-related differences in measured cognitive abilities seem to accumulate during the first two years of primary education.

Discovering the early determinants of ability formation is crucial if the development of cognitive skills and personality traits indeed depends on the stock of previously acquired skills, as suggested by [14] and [19]. Improving the quality of the early childhood environment potentially decreases outcome inequality in earlier and later stages of the lifecycle at a lower cost when compared to interventions that target older age groups.

From the results of this paper, we can derive a number of policy implications. First, lower-achieving schools might require closer monitoring since their students appear to develop cognitive abilities more slowly than those in high-achieving schools. Second, since it has been documented that some parents seem to have weak preferences for school quality (see [5]), more children may benefit from attending higher quality schools if parents are more aware of quality differences that exist between the schools that they can choose from.

The exact channels through which the observed relationship operates, however, is not yet clearly identified. Whether teachers, peers, the educational concept or other unobserved factors that relate to school quality drive the results remains subject to future research.

Policy makers—especially in the US—have been experimenting with the introduction of different kinds of school choice programs. Parents are increasingly given the opportunity to choose schools with higher average student achievement scores. The introduction of these school choice policies is based on the implicit assumptions that (1) parents will choose better schools if they are given the opportunity (see [5]) and that (2) there is a link between school quality and individual student performance. Our study provides evidence that the latter assumption seems to hold in the well-established education system of free school choice in the Netherlands. Our findings indicate that the relationship between school quality and cognitive development is already visible during the first two years of preschool.

A limitation of our study is that although we take a number of important measures to increase the likelihood that our results have a causal interpretation, it is not possible to erase all doubt about unobserved confounding factors. Given the measures we take, it is implausible that remaining bias will substantially affect our results. Firstly, we do not look at levels of cognitive skills, but rather we look at changes in these skills by controlling for initial level of measured cognitive ability. This should take sorting into different schools based on initial skills into account. Secondly, since children’s cognitive development may also be affected by parental investments and the socio-economic background of parents, we control for the education level of the mother and father as well as the household income. Thirdly, since access to above median quality schools may be affected by neighborhood characteristics, we show that our results are robust to the inclusion of a set of neighborhood characteristics: the percentage of households under the social minimum income, the percentage of households with low income (less than €25,100 per year), the percentage of households with high income (more than €46,500 per year), and the percentage of households with at least one child. Fourthly, we show that results are robust to the inclusion of neighborhood fixed effects (see S10, S11 and S12 Tables). Fifthly, in the robustness section, we perform an instrumental variable approach. The identifying assumption is that conditional on the large set of included individual and neighborhood controls, proximity to a higher achieving school is unrelated to unobservables which may also affect test scores. The coefficient remains positive and significant (even increases) when we control for many potentially important observables, and when we use an IV approach. Therefore, it appears implausible that unobserved factors could drive down the results in any substantial way.

## Supporting Information

S1 Table Determinants of baseline ability. (DOCX)

S2 Table Full version of table 3 displaying all included controls. (DOCX)

S3 Table Full version of table 4 displaying all included controls. Gender differences in early cognitive development.
(DOCX)

S4 Table Gender differences in early cognitive development. (DOCX)

S5 Table Alternative versions of Table 4 and Table 5 for the survey sample without controls vs. full sample without controls. (DOCX)

S6 Table Full version of table 6 displaying all included controls. (DOCX)

S7 Table Alternative version of table 4 using a continuous CITO model—OLS estimation. (DOCX)

S8 Table Alternative version of table 6 using a continuous CITO model—IV estimation. (DOCX)

S9 Table Observable characteristics and proximity to higher-achieving schools. (DOCX)

S10 Table Robustness check: Table 3 including neighborhood fixed effects. (DOCX)

S11 Table Robustness check: Table 4 including neighborhood fixed effects. (DOCX)

S12 Table Robustness check: Table 6 IV estimation including neighborhood fixed effects. (DOCX)

S1 Text Supporting information about the cognitive achievement tests. Examples and description of the test.
(DOCX)
