# Supplementary material for: School Quality and the Development of Cognitive Skills between Age Four and Six
Source: PLoS One. 2015 Jul 16;10(7):e0129700. doi: 10.1371/journal.pone.0129700 (PMC4504490; doi:10.1371/journal.pone.0129700)
Supplement: S1 Table — (DOCX) [file pone.0129700.s001.docx]

**S1 Table. Determinants of baseline ability**

|  | (1) | (2) | (3) |  |
| --- | --- | --- | --- | --- |
|  | Test 1 | Test 1 | Test 1 |  |
|  | OLS | OLS | OLS |  |
|  |  |  |  |  |
| Higher-achieving school | 0.173*** | 0.112** | 0.088 |  |
|  | (0.052) | (0.053) | (0.054) |  |
| **Household characteristics** |  |  |  |  |
| Education level mother middle |  | 0.212** | 0.204* |  |
|  |  | (0.104) | (0.104) |  |
| Education level mother high |  | 0.465*** | 0.447*** |  |
|  |  | (0.121) | (0.121) |  |
| Mother education level missing |  | -0.062 | -0.067 |  |
|  |  | (0.186) | (0.186) |  |
| Education level father middle |  | 0.009 | 0.006 |  |
|  |  | (0.098) | (0.099) |  |
| Education level father high |  | 0.043 | 0.034 |  |
|  |  | (0.110) | (0.110) |  |
| Father education level missing |  | 0.111 | 0.111 |  |
|  |  | (0.172) | (0.172) |  |
| Income 1501 - 2500 € |  | 0.174 | 0.162 |  |
|  |  | (0.126) | (0.126) |  |
| Income 2501 - 3500 € |  | 0.156 | 0.135 |  |
|  |  | (0.123) | (0.123) |  |
| Income 3501 - 4500 € |  | 0.275* | 0.255* |  |
|  |  | (0.144) | (0.144) |  |
| Income above 4501 |  | 0.162 | 0.148 |  |
|  |  | (0.150) | (0.150) |  |
| Income information missing |  | 0.289** | 0.270** |  |
|  |  | (0.123) | (0.124) |  |
| **Neighborhood characteristics** |  |  |  |  |
| % HH under the social minimum |  |  | -0.007 |  |
|  |  |  | (0.015) |  |
| % HH with low income |  |  | 0.002 |  |
|  |  |  | (0.008) |  |
| % HH with high income |  |  | 0.009 |  |
|  |  |  | (0.008) |  |
| % HH with one or more children |  |  | -0.002 |  |
|  |  |  | (0.006) |  |
| Constant | -0.023 | -0.410*** | -0.495 |  |
|  | (0.037) | (0.122) | (0.474) |  |
|  |  |  |  |  |
| Observations | 1,112 | 1,112 | 1,112 |  |
| Adj. R-squared | 0.00907 | 0.0355 | 0.0357 |  |

Notes: The dependent variable, the score on test 1, is standardized to mean zero and a standard deviation of one. A higher-achieving school is defined as having an above median three year school average CITO score. Neighborhood characteristics were measured at the four digit postal code area by CBS Statistics Netherlands. The omitted education categories are “Education level mother low” and “Education level father low”. The omitted monthly household income category is “Income below € 1,500”. Standard errors are in parentheses; *** p<0.01, ** p<0.05, * p<0.1.
