# Supplementary material for: School Quality and the Development of Cognitive Skills between Age Four and Six
Source: PLoS One. 2015 Jul 16;10(7):e0129700. doi: 10.1371/journal.pone.0129700 (PMC4504490; doi:10.1371/journal.pone.0129700)
Supplement: S2 Table — (DOCX) [file pone.0129700.s002.docx]

**S2 Table. Full version of table 3 displaying all included controls**

|  | (1) | (2) | (3) |  | (4) | (5) | (6) |
| --- | --- | --- | --- | --- | --- | --- | --- |
|  | Test 2 | Test 3 | Test 4 |  | Test 2 | Test 3 | Test 4 |
|  |  |  |  |  |  |  |  |
| Higher achieving school | 0.040 | 0.163*** | 0.177*** |  | 0.056 | 0.153*** | 0.167*** |
|  | (0.036) | (0.046) | (0.040) |  | (0.037) | (0.048) | (0.041) |
| Test 1 | 0.671*** | 0.599*** | 0.521*** |  | 0.673*** | 0.598*** | 0.519*** |
|  | (0.020) | (0.027) | (0.023) |  | (0.020) | (0.027) | (0.023) |
| Time between test 1 & 2 (in months) | 0.001 |  |  |  | -0.004 |  |  |
|  | (0.024) |  |  |  | (0.024) |  |  |
| Time between test 1 & 3 (in months) |  | 0.051* |  |  |  | 0.050* |  |
|  |  | (0.027) |  |  |  | (0.027) |  |
| Time between test 1 & 4 (in months) |  |  | 0.035** |  |  |  | 0.038** |
|  |  |  | (0.017) |  |  |  | (0.017) |
| Mother: No degree | -0.205 | -0.011 | 0.254 |  | -0.207 | 0.003 | 0.268 |
|  | (0.146) | (0.191) | (0.165) |  | (0.146) | (0.191) | (0.165) |
| Mother: Lower vocational education | -0.113 | 0.027 | 0.122 |  | -0.110 | 0.029 | 0.114 |
|  | (0.134) | (0.175) | (0.151) |  | (0.134) | (0.175) | (0.151) |
| Mother: General continued education | -0.144 | 0.201 | 0.287** |  | -0.135 | 0.212 | 0.288** |
|  | (0.126) | (0.165) | (0.142) |  | (0.126) | (0.164) | (0.142) |
| Mother: Preparatory scientific education | -0.053 | 0.081 | 0.229* |  | -0.043 | 0.100 | 0.235* |
|  | (0.118) | (0.154) | (0.133) |  | (0.118) | (0.154) | (0.133) |
| Mother: Higher professional education | 0.046 | 0.198 | 0.265* |  | 0.057 | 0.209 | 0.272* |
|  | (0.126) | (0.164) | (0.142) |  | (0.126) | (0.164) | (0.142) |
| Mother: University degree | -0.074 | 0.197 | 0.285* |  | -0.062 | 0.180 | 0.271* |
|  | (0.144) | (0.189) | (0.163) |  | (0.145) | (0.189) | (0.163) |
| Father: No degree | 0.054 | -0.098 | -0.266* |  | 0.040 | -0.111 | -0.270* |
|  | (0.136) | (0.177) | (0.153) |  | (0.136) | (0.177) | (0.153) |
| Father: Lower vocational education | -0.176 | -0.195 | -0.281** |  | -0.179 | -0.205 | -0.297** |
|  | (0.122) | (0.159) | (0.137) |  | (0.122) | (0.159) | (0.137) |
| Father: General continued education | -0.030 | -0.162 | -0.166 |  | -0.037 | -0.160 | -0.163 |
|  | (0.129) | (0.168) | (0.145) |  | (0.129) | (0.168) | (0.145) |
| Father: Preparatory scientific education | 0.059 | -0.009 | -0.132 |  | 0.057 | -0.019 | -0.146 |
|  | (0.115) | (0.151) | (0.130) |  | (0.115) | (0.151) | (0.130) |
| Father: Higher professional education | 0.156 | 0.015 | 0.007 |  | 0.150 | -0.009 | -0.012 |
|  | (0.120) | (0.156) | (0.135) |  | (0.120) | (0.157) | (0.135) |
| Father: University degree | 0.120 | -0.010 | -0.024 |  | 0.118 | -0.041 | -0.054 |
|  | (0.130) | (0.170) | (0.147) |  | (0.131) | (0.170) | (0.147) |
| Income: below 800 | 0.097 | -0.262 | 0.008 |  | 0.079 | -0.260 | 0.015 |
|  | (0.172) | (0.224) | (0.194) |  | (0.172) | (0.224) | (0.194) |
| Income: 800- 1250 | 0.042 | -0.134 | -0.229** |  | 0.034 | -0.129 | -0.218** |
|  | (0.088) | (0.115) | (0.099) |  | (0.088) | (0.115) | (0.100) |
| Income: 1250 - 1750 | 0.013 | 0.025 | -0.069 |  | 0.004 | 0.018 | -0.060 |
|  | (0.086) | (0.112) | (0.097) |  | (0.086) | (0.112) | (0.097) |
| Income: 1750 - 2250 | 0.023 | 0.079 | -0.034 |  | 0.017 | 0.081 | -0.028 |
|  | (0.081) | (0.106) | (0.092) |  | (0.081) | (0.106) | (0.092) |
| Income: 2250 - 2750 | 0.074 | 0.150 | 0.001 |  | 0.074 | 0.140 | -0.001 |
|  | (0.075) | (0.099) | (0.085) |  | (0.075) | (0.098) | (0.085) |
| Income: 2750 - 3250 | -0.006 | 0.055 | 0.027 |  | -0.004 | 0.062 | 0.032 |
|  | (0.074) | (0.096) | (0.083) |  | (0.074) | (0.096) | (0.083) |
| Income: 3250 - 3750 | -0.018 | -0.088 | -0.219** |  | -0.022 | -0.101 | -0.224** |
|  | (0.088) | (0.115) | (0.099) |  | (0.088) | (0.115) | (0.099) |
| Income: 3750 - 4250 | -0.004 | 0.146 | 0.003 |  | -0.002 | 0.161 | 0.019 |
|  | (0.111) | (0.146) | (0.126) |  | (0.111) | (0.146) | (0.126) |
| Income: 4250 - 4750 | 0.014 | 0.121 | -0.027 |  | 0.014 | 0.114 | -0.027 |
|  | (0.127) | (0.166) | (0.143) |  | (0.127) | (0.166) | (0.143) |
| Income: 4750 - 5250 | 0.058 | 0.080 | 0.098 |  | 0.042 | 0.069 | 0.106 |
|  | (0.128) | (0.168) | (0.145) |  | (0.129) | (0.168) | (0.145) |
| Income: above 5250 | 0.018 | 0.110 | 0.049 |  | 0.009 | 0.102 | 0.055 |
|  | (0.111) | (0.145) | (0.125) |  | (0.111) | (0.145) | (0.125) |
| % HH under the social minimum |  |  |  |  | 0.019* | 0.028** | 0.017 |
|  |  |  |  |  | (0.010) | (0.013) | (0.012) |
| % HH with low income |  |  |  |  | -0.006 | -0.010 | -0.012** |
|  |  |  |  |  | (0.005) | (0.007) | (0.006) |
| % HH with high income |  |  |  |  | -0.004 | 0.007 | -0.001 |
|  |  |  |  |  | (0.005) | (0.007) | (0.006) |
| % HH with one or more children |  |  |  |  | 0.000 | -0.008 | -0.006 |
|  |  |  |  |  | (0.004) | (0.005) | (0.004) |
| Constant | 0.078 | -0.756** | -0.645** |  | 0.236 | -0.406 | -0.086 |
|  | (0.100) | (0.315) | (0.270) |  | (0.331) | (0.481) | (0.437) |
|  |  |  |  |  |  |  |  |
| Observations | 1,112 | 1,112 | 1,112 |  | 1,112 | 1,112 | 1,112 |
| R-squared | 0.539 | 0.381 | 0.394 |  | 0.541 | 0.385 | 0.398 |
| Adj. R-squared | 0.528 | 0.366 | 0.379 |  | 0.528 | 0.368 | 0.381 |

Notes: All test scores are standardized to mean zero and a standard deviation of one. A higher-achieving school is defined as having an above median three year school average CITO score. Parental background controls are the household income and the education level of the father and mother. Omitted category is rejected to answer sub-question. Neighborhood controls include a set of variables measured at the four digit postal code area. The neighborhood controls are the percentage of households under the social minimum income, the percentage of households with low income (less than €25,100 per year), the percentage of households with high income (more than €46,500 per year) and the percentage of households with at least one child. The data on neighborhood characteristics was collected by CBS Statistics Netherlands. Standard errors are in parentheses; *** p<0.01, ** p<0.05, * p<0.1.
