# Supplementary material for: School Quality and the Development of Cognitive Skills between Age Four and Six
Source: PLoS One. 2015 Jul 16;10(7):e0129700. doi: 10.1371/journal.pone.0129700 (PMC4504490; doi:10.1371/journal.pone.0129700)
Supplement: S5 Table — (DOCX) [file pone.0129700.s005.docx]

**S5 Table. Alternative versions of Table 4 and Table 5 for the survey sample without controls vs. full sample without controls**

| **Panel A (ref. Table 4)** | Survey sample without controls | | |  | Full sample without controls | | |
| --- | --- | --- | --- | --- | --- | --- | --- |
|  | (1) | (2) | (3) |  | (4) | (5) | (6) |
|  | Test 2 | Test 3 | Test 4 |  | Test 2 | Test 3 | Test 4 |
|  |  |  |  |  |  |  |  |
| Higher achieving school | 0.066* | 0.212*** | 0.213*** |  | 0.058* | 0.207*** | 0.162*** |
|  | (0.035) | (0.045) | (0.039) |  | (0.034) | (0.043) | (0.038) |
| Test 1 | 0.686*** | 0.618*** | 0.539*** |  | 0.677*** | 0.597*** | 0.501*** |
|  | (0.020) | (0.026) | (0.023) |  | (0.019) | (0.024) | (0.021) |
| Time between test 1 & 2 (in months) | 0.001 |  |  |  | 0.047*** |  |  |
|  | (0.024) |  |  |  | (0.016) |  |  |
| Time between test 1 & 3 (in months) |  | 0.060** |  |  |  | 0.075*** |  |
|  |  | (0.026) |  |  |  | (0.008) |  |
| Time between test 1 & 4 (in months) |  |  | 0.039** |  |  |  | 0.038*** |
|  |  |  | (0.017) |  |  |  | (0.007) |
| Constant | 0.074 | -0.826*** | -0.661** |  | -0.131* | -0.988*** | -0.591*** |
|  | (0.097) | (0.313) | (0.270) |  | (0.071) | (0.107) | (0.110) |
|  |  |  |  |  |  |  |  |
| Observations | 1,112 | 1,112 | 1,112 |  | 1,299 | 1,299 | 1,299 |
| R-squared | 0.523 | 0.361 | 0.367 |  | 0.511 | 0.349 | 0.324 |
| Adj. R-squared | 0.522 | 0.359 | 0.365 |  | 0.510 | 0.347 | 0.322 |
|  |  |  |  |  |  |  |  |
| **Panel B (ref. Table 5)** | Survey sample without controls | | |  | Full sample without controls | | |
|  | (1) | (2) | (3) |  | (4) | (5) | (6) |
|  | Test 2 | Test 3 | Test 4 |  | Test 2 | Test 3 | Test 4 |
|  |  |  |  |  |  |  |  |
| Higher achieving school | 0.070** | 0.214*** | 0.218*** |  | 0.058* | 0.207*** | 0.162*** |
|  | (0.035) | (0.046) | (0.039) |  | (0.034) | (0.043) | (0.038) |
| Higher achieving school * Test 1 | -0.054 | -0.026 | -0.070 |  | -0.048 | -0.012 | -0.002 |
|  | (0.040) | (0.052) | (0.045) |  | (0.037) | (0.048) | (0.042) |
| Test 1 | 0.713*** | 0.631*** | 0.575*** |  | 0.700*** | 0.603*** | 0.502*** |
|  | (0.028) | (0.037) | (0.032) |  | (0.026) | (0.034) | (0.029) |
| Time between test 1 & 2 (in months) | -0.002 |  |  |  | 0.048*** |  |  |
|  | (0.024) |  |  |  | (0.016) |  |  |
| Time between test 1 & 3 (in months) |  | 0.060** |  |  |  | 0.075*** |  |
|  |  | (0.026) |  |  |  | (0.008) |  |
| Time between test 1 & 4 (in months) |  |  | 0.038** |  |  |  | 0.038*** |
|  |  |  | (0.017) |  |  |  | (0.007) |
| Constant | 0.085 | -0.818*** | -0.648** |  | -0.131* | -0.990*** | -0.591*** |
|  | (0.097) | (0.313) | (0.270) |  | (0.071) | (0.108) | (0.110) |
|  |  |  |  |  |  |  |  |
| Observations | 1,112 | 1,112 | 1,112 |  | 1,299 | 1,299 | 1,299 |
| Adj. R-squared | 0.522 | 0.359 | 0.366 |  | 0.511 | 0.347 | 0.322 |

Notes: All test scores are standardized to mean zero and a standard deviation of one. A higher-achieving school is defined as having an above median three year school average CITO score. Standard errors are in parentheses; *** p<0.01, ** p<0.05, * p<0.1.
