# Supplementary material for: School Quality and the Development of Cognitive Skills between Age Four and Six
Source: PLoS One. 2015 Jul 16;10(7):e0129700. doi: 10.1371/journal.pone.0129700 (PMC4504490; doi:10.1371/journal.pone.0129700)
Supplement: S7 Table — (DOCX) [file pone.0129700.s007.docx]

**S7 Table. Alternative version of table 4 using a continuous CITO model – OLS estimation**

|  | (1) | (2) | (3) | (4) | (5) | (6) |
| --- | --- | --- | --- | --- | --- | --- |
|  | Test 2 | Test 3 | Test 4 | Test 2 | Test 3 | Test 4 |
|  |  |  |  |  |  |  |
| Std. school CITO | -0.011 | 0.086*** | 0.098*** | -0.000 | 0.082*** | 0.090*** |
|  | (0.020) | (0.025) | (0.022) | (0.021) | (0.027) | (0.023) |
| Test 1 | 0.672*** | 0.607*** | 0.521*** | 0.673*** | 0.607*** | 0.521*** |
|  | (0.021) | (0.027) | (0.023) | (0.021) | (0.027) | (0.023) |
| Time between test 1 & 2 (in months) | 0.009 |  |  | 0.001 |  |  |
|  | (0.028) |  |  | (0.028) |  |  |
| Time between test 1 & 3 (in months) |  | 0.052* |  |  | 0.052* |  |
|  |  | (0.027) |  |  | (0.027) |  |
| Time between test 1 & 4 (in months) |  |  | 0.035** |  |  | 0.038** |
|  |  |  | (0.017) |  |  | (0.017) |
| Mother: No degree | -0.199 | 0.061 | 0.323* | -0.203 | 0.072 | 0.332** |
|  | (0.149) | (0.193) | (0.167) | (0.149) | (0.193) | (0.167) |
| Mother: Lower vocational education | -0.130 | -0.017 | 0.072 | -0.130 | -0.010 | 0.071 |
|  | (0.136) | (0.176) | (0.152) | (0.136) | (0.176) | (0.152) |
| Mother: General continued education | -0.131 | 0.150 | 0.253* | -0.127 | 0.167 | 0.258* |
|  | (0.128) | (0.166) | (0.143) | (0.128) | (0.165) | (0.143) |
| Mother: Preparatory scientific education | -0.049 | 0.043 | 0.188 | -0.044 | 0.065 | 0.197 |
|  | (0.119) | (0.154) | (0.133) | (0.119) | (0.154) | (0.133) |
| Mother: Higher professional education | 0.046 | 0.154 | 0.223 | 0.049 | 0.169 | 0.234 |
|  | (0.127) | (0.165) | (0.142) | (0.128) | (0.165) | (0.143) |
| Mother: University degree | -0.096 | 0.144 | 0.251 | -0.093 | 0.128 | 0.242 |
|  | (0.146) | (0.189) | (0.164) | (0.146) | (0.189) | (0.164) |
| Father: No degree | 0.067 | -0.109 | -0.293* | 0.056 | -0.127 | -0.300* |
|  | (0.138) | (0.178) | (0.154) | (0.138) | (0.178) | (0.154) |
| Father: Lower vocational education | -0.154 | -0.145 | -0.230* | -0.155 | -0.153 | -0.242* |
|  | (0.123) | (0.159) | (0.138) | (0.123) | (0.159) | (0.138) |
| Father: General continued education | -0.025 | -0.139 | -0.146 | -0.029 | -0.141 | -0.146 |
|  | (0.131) | (0.169) | (0.146) | (0.131) | (0.169) | (0.146) |
| Father: Preparatory scientific education | 0.065 | 0.020 | -0.121 | 0.067 | 0.016 | -0.128 |
|  | (0.116) | (0.150) | (0.130) | (0.116) | (0.151) | (0.130) |
| Father: Higher professional education | 0.177 | 0.050 | 0.018 | 0.173 | 0.031 | 0.006 |
|  | (0.121) | (0.157) | (0.135) | (0.121) | (0.157) | (0.136) |
| Father: University degree | 0.140 | -0.034 | -0.059 | 0.139 | -0.056 | -0.079 |
|  | (0.133) | (0.172) | (0.148) | (0.133) | (0.172) | (0.149) |
| Income: below 800 | 0.062 | -0.425* | -0.149 | 0.056 | -0.434* | -0.155 |
|  | (0.187) | (0.243) | (0.210) | (0.188) | (0.242) | (0.210) |
| Income: 800- 1250 | 0.041 | -0.072 | -0.197* | 0.037 | -0.071 | -0.192* |
|  | (0.090) | (0.117) | (0.101) | (0.090) | (0.116) | (0.101) |
| Income: 1250 - 1750 | 0.005 | 0.042 | -0.046 | -0.002 | 0.031 | -0.042 |
|  | (0.086) | (0.112) | (0.097) | (0.087) | (0.112) | (0.097) |
| Income: 1750 - 2250 | 0.010 | 0.097 | -0.006 | 0.007 | 0.096 | -0.003 |
|  | (0.084) | (0.108) | (0.094) | (0.084) | (0.108) | (0.094) |
| Income: 2250 - 2750 | 0.071 | 0.158 | 0.015 | 0.070 | 0.144 | 0.011 |
|  | (0.077) | (0.099) | (0.086) | (0.077) | (0.099) | (0.086) |
| Income: 2750 - 3250 | -0.004 | 0.043 | 0.041 | -0.001 | 0.048 | 0.045 |
|  | (0.075) | (0.097) | (0.084) | (0.075) | (0.097) | (0.084) |
| Income: 3250 - 3750 | -0.013 | -0.062 | -0.181* | -0.018 | -0.077 | -0.186* |
|  | (0.090) | (0.116) | (0.100) | (0.090) | (0.116) | (0.100) |
| Income: 3750 - 4250 | 0.004 | 0.158 | 0.019 | 0.003 | 0.173 | 0.034 |
|  | (0.112) | (0.145) | (0.126) | (0.113) | (0.145) | (0.126) |
| Income: 4250 - 4750 | 0.021 | 0.105 | -0.035 | 0.020 | 0.090 | -0.042 |
|  | (0.132) | (0.171) | (0.147) | (0.132) | (0.171) | (0.148) |
| Income: 4750 - 5250 | 0.064 | 0.114 | 0.134 | 0.049 | 0.094 | 0.135 |
|  | (0.130) | (0.168) | (0.145) | (0.130) | (0.168) | (0.146) |
| Income: above 5250 | 0.018 | 0.165 | 0.106 | 0.010 | 0.147 | 0.106 |
|  | (0.114) | (0.147) | (0.127) | (0.114) | (0.147) | (0.127) |
| % HH under the social minimum |  |  |  | 0.016 | 0.029** | 0.015 |
|  |  |  |  | (0.010) | (0.014) | (0.012) |
| % HH with low income |  |  |  | -0.004 | -0.010 | -0.010* |
|  |  |  |  | (0.005) | (0.007) | (0.006) |
| % HH with high income |  |  |  | -0.002 | 0.006 | -0.001 |
|  |  |  |  | (0.006) | (0.007) | (0.006) |
| % HH with one or more children |  |  |  | -0.000 | -0.010* | -0.007 |
|  |  |  |  | (0.004) | (0.005) | (0.004) |
| Constant | 0.059 | -0.687** | -0.539** | 0.188 | -0.234 | -0.015 |
|  | (0.120) | (0.318) | (0.272) | (0.344) | (0.484) | (0.440) |
|  |  |  |  |  |  |  |
| Observations | 1,076 | 1,076 | 1,076 | 1,076 | 1,076 | 1,076 |
| R-squared | 0.536 | 0.384 | 0.391 | 0.537 | 0.389 | 0.394 |

Note: The data on neighborhood characteristics was collected by CBS Statistics Netherlands. Standard errors are in parentheses; *** p<0.01, ** p<0.05, * p<0.1
