# Supplementary material for: School Quality and the Development of Cognitive Skills between Age Four and Six
Source: PLoS One. 2015 Jul 16;10(7):e0129700. doi: 10.1371/journal.pone.0129700 (PMC4504490; doi:10.1371/journal.pone.0129700)
Supplement: S9 Table — (DOCX) [file pone.0129700.s009.docx]

**S9 Table. Observable characteristics and proximity to higher-achieving schools**

|  | (1) |
| --- | --- |
|  | One if closest school is higher-achieving |
|  |  |
| Test 1 | -0.000 |
|  | (0.022) |
| Test 2 | -0.013 |
|  | (0.024) |
| Test 3 | 0.049** |
|  | (0.021) |
| Test 4 | 0.040* |
|  | (0.024) |
| Mother: No degree | -0.026 |
|  | (0.110) |
| Mother: Lower vocational education | 0.056 |
|  | (0.106) |
| Mother: General continued education | 0.073 |
|  | (0.100) |
| Mother: Preparatory scientific education | 0.072 |
|  | (0.094) |
| Mother: Higher professional education | 0.174* |
|  | (0.100) |
| Mother: University degree | 0.146 |
|  | (0.115) |
| Father: No degree | 0.011 |
|  | (0.106) |
| Father: Lower vocational education | 0.014 |
|  | (0.097) |
| Father: General continued education | -0.085 |
|  | (0.102) |
| Father: Preparatory scientific education | 0.001 |
|  | (0.092) |
| Father: Higher professional education | 0.040 |
|  | (0.095) |
| Father: University degree | 0.017 |
|  | (0.104) |
| Income: below 800 | -0.076 |
|  | (0.135) |
| Income: 800- 1250 | -0.130* |
|  | (0.069) |
| Income: 1250 - 1750 | -0.107 |
|  | (0.067) |
| Income: 1750 - 2250 | 0.016 |
|  | (0.062) |
| Income: 2250 - 2750 | 0.098* |
|  | (0.059) |
| Income: 2750 - 3250 | 0.088 |
|  | (0.057) |
| Income: 3250 - 3750 | 0.062 |
|  | (0.070) |
| Income: 3750 - 4250 | 0.078 |
|  | (0.083) |
| Income: 4250 - 4750 | 0.251** |
|  | (0.100) |
| Income: 4750 - 5250 | 0.093 |
|  | (0.103) |
| Income: above 5250 | -0.021 |
|  | (0.088) |
| Constant | 0.468*** |
|  | (0.023) |
|  |  |
| Observations | 1,299 |
| Adj. R-squared | 0.0670 |

Note: Standard errors are in parentheses; *** p<0.01, ** p<0.05, * p<0.1
