# Supplementary material for: School Quality and the Development of Cognitive Skills between Age Four and Six
Source: PLoS One. 2015 Jul 16;10(7):e0129700. doi: 10.1371/journal.pone.0129700 (PMC4504490; doi:10.1371/journal.pone.0129700)
Supplement: S12 Table — (DOCX) [file pone.0129700.s012.docx]

**S12 Table. Robustness check: Table 6 IV estimation including neighborhood fixed effects**

|  | (1) | (2) | (3) |  | (4) | (5) | (6) |
| --- | --- | --- | --- | --- | --- | --- | --- |
|  | Test 2 | Test 3 | Test 4 |  | Test 2 | Test 3 | Test 4 |
|  |  |  |  |  |  |  |  |
| Std. school CITO | -0.004 | 0.080*** | 0.092*** |  | -0.045 | 0.068 | 0.079** |
|  | (0.021) | (0.027) | (0.023) |  | (0.037) | (0.045) | (0.040) |
| Test 1 | 0.678*** | 0.603*** | 0.520*** |  | 0.666*** | 0.590*** | 0.508*** |
|  | (0.020) | (0.027) | (0.023) |  | (0.022) | (0.028) | (0.024) |
| Time between test 1 & 2 (in months) | 0.008 |  |  |  | 0.012 |  |  |
|  | (0.024) |  |  |  | (0.030) |  |  |
| Time between test 1 & 3 (in months) |  | 0.050* |  |  |  | 0.109*** |  |
|  |  | (0.027) |  |  |  | (0.034) |  |
| Time between test 1 & 4 (in months) |  |  | 0.039** |  |  |  | 0.020 |
|  |  |  | (0.017) |  |  |  | (0.021) |
| Constant | 0.177 | -0.331 | -0.009 |  | 0.048 | -1.337*** | -0.282 |
|  | (0.333) | (0.483) | (0.438) |  | (0.129) | (0.401) | (0.333) |
|  |  |  |  |  |  |  |  |
| Observations | 1,106 | 1,106 | 1,106 |  | 1,106 | 1,106 | 1,106 |
| R-squared | 0.542 | 0.386 | 0.396 |  | 0.585 | 0.495 | 0.492 |
| Parental background controls | Yes | Yes | Yes |  | Yes | Yes | Yes |
| Neighborhood controls | Yes | Yes | Yes |  | - | - | - |
| Neighborhood fixed effects | No | No | No |  | Yes | Yes | Yes |

Note: Parental background controls are the household income and the education level of the father and mother. Neighborhood controls include a set of variables measured at the four digit postal code area. The neighborhood controls are the percentage of households under the social minimum income, the percentage of households with low income (less than €25,100 per year), the percentage of households with high income (more than €46,500 per year) and the percentage of households with at least one child. The data on neighborhood characteristics was collected by CBS Statistics Netherlands. Standard errors are in parentheses; *** p<0.01, ** p<0.05, * p<0.1
