# Supplementary material for: School Quality and the Development of Cognitive Skills between Age Four and Six
Source: PLoS One. 2015 Jul 16;10(7):e0129700. doi: 10.1371/journal.pone.0129700 (PMC4504490; doi:10.1371/journal.pone.0129700)
Supplement: S1 Text — Examples and description of the test. (DOCX) [file pone.0129700.s013.docx]

**S1 Text. Supporting information about the cognitive achievement tests. Examples and description of the test.**

[*see attached image file* S13 fig]

“Ordering test for kindergarten children” **(***“Ordenen voor jongste kleuters”*) The text was read out aloud to the children, and the child had to mark the respective box in their booklet. Teachers were instructed to repeat the item only once if necessary. Not answering a question was counted as a wrong answer. Every test had 42 items. The following questions are translated from Dutch.
